# Postoperative neuroimaging analysis of DRT deep brain stimulation revision surgery for complicated essential tremor

**DOI:** 10.1007/s00701-017-3134-z

**Published:** 2017-03-10

**Authors:** Volker Arnd Coenen, Balint Varkuti, Yaroslav Parpaley, Sabine Skodda, Thomas Prokop, Horst Urbach, Meng Li, Peter Christoph Reinacher

**Affiliations:** 1 Department of Stereotactic and Functional Neurosurgery, Medical Faculty, Freiburg University Medical Center, Freiburg (i.Br.), Germany; 2 Brainlab AG, Feldkirchen, Germany; 3 Department of Neurosurgery, University Hospital Knappschaftskrankenhaus, Ruhr University Bochum, Bochum, Germany; 4 Department of Neurology, University Hospital Knappschaftskrankenhaus, Ruhr-University Bochum, Bochum, Germany; 5 Department of Neuroradiology, Freiburg University Medical Center, Freiburg (i.Br.), Germany

**Keywords:** Deep brain stimulation, Dentato-rubro-thalamic tract, Diffusion tensor imaging, Essential tremor, Fiber tracking, STN, Thalamus, Tractography

## Abstract

**Background:**

We report a patient who received conventional bilateral deep brain stimulation of the ventral intermediate nucleus of thalamus (Vim) for the treatment of medication refractory essential tremor (ET). After initial beneficial effects, therapeutic efficacy was lost due to a loss of control of his proximal trunkal and extremity tremor. The patient received successful diffusion tensor magnetic resonance imaging fiber tractographic (DTI FT)-assisted DBS revision surgery targeting the dentato-rubro-thalamic tract (DRT) in the subthalamic region (STR).

**Objective:**

To report the concept of DTI FT-assisted DRT DBS revision surgery for ET and to show sophisticated postoperative neuroimaging analysis explaining improved symptom control.

**Methods:**

Analysis was based on preoperative DTI sequences and postoperative helical computed tomography (hCT). Leads, stimulation fields, and fibers were reconstructed using commercial software systems (Elements, Brainlab AG, Feldkirchen, Germany; GUIDE XT, Boston Scientific Corp., Boston, MA, USA).

**Results:**

The patient showed immediate and sustained tremor improvement after DTI FT-assisted revision surgery. Analysis of the two implantations (electrode positions in both instances) revealed a lateral and posterior shift in the pattern of modulation of the cortical fiber pathway projection after revision surgery as compared to initial implantation, explaining a more efficacious stimulation.

**Conclusions:**

Our work underpins a possible superiority of direct targeting approaches using advanced neuroimaging technologies to perform personalized DBS surgery. The evaluation of DBS electrode positions with the herein-described neuroimaging simulation technologies will likely improve targeting and revision strategies. Direct targeting with DTI FT-assisted approaches in a variety of indications is the focus of our ongoing research.

## Introduction

Deep brain stimulation (DBS) has become a standardized treatment for medically refractory essential tremor (ET) despite a current renaissance of lesion surgery by means of focused ultrasonography [8, 9, 13, 18]. Diffusion tensor magnetic resonance imaging fiber tractographic (DTI FT)-assisted DBS allows for directly targeting the dentato-rubro-thalamic tract, which has been identified as part of the tremor-promoting cerebello-thalamo-cortical network [4] and as a target structure for DBS. After introduction of this targeting technology in 2011 [3, 7], other groups have now started to evaluate and report this approach [21], which is increasingly recognized as part of the armamentarium of DBS and lesion surgery for tremor [12, 23]. For a review of this topic, please refer to [2].

We here report a case of a patient who received bilateral conventional deep brain stimulation of the ventral intermediate nucleus of thalamus (Vim) for the treatment of extremely debilitating refractory essential tremor (ET). After initial beneficial effects, therapeutic efficacy was lost due to a loss of control of his proximal trunkal tremor. The patient received successful diffusion tensor magnetic resonance imaging fiber tractographic (DTI FT)-assisted DBS revision surgery targeting the dentato-rubro-thalamic tract (DRT) in the subthalamic region.

## Case illustration

A 72-year-old male patient had been treated with Vim DBS for an extremely constraining essential tremor (ET) predominantly involving the upper extremities. At the same time, he showed a marked proximal and trunkal “yes-yes”-tremor especially in the upright position interfering with his gait. He was identified as a candidate for Vim DBS surgery according to consensus guidelines [8]. Preoperative (before DBS#1), the essential tremor rating scale (ETRS) score was 60. Intraoperative effects were promising. Typical implantation in the Vim region was performed with an Activa RC system (Medtronic, USA) and bilateral DBS electrodes (model 3389, Medtronic, USA). Initial tremor suppression was promising under the micro-lesioning effect. Over the first weeks, stimulation amplitudes had to be escalated. There was sufficient control over the right upper extremity and somewhat less effective tremor suppression in the left upper extremity. After the micro-lesioning effect subsided completely, trunk tremor and proximal extremity tremor reoccurred. Higher stimulation amplitudes led to marked ataxia and gait disturbance. The postoperative ETRS score was 58, indicating no improvement. Adjustments over time led to no marked improvement. Considering the potential effect of DBS in the subthalamic region (STR, CZi) on the proximal residual symptoms and the absence of diffusion imaging in the initial implantation, we suggested to replace the whole DBS system and implant new DBS electrodes in the subthalamic region [11, 16, 17] with the assistance of DTI FT.

## Methods

### Ethics

Freiburg University’s institutional review board granted the presentation of DTI fiber tractographic results together with clinical results (No. 567/14). The patient gave informed consent as to publication of this data. The study followed the tenets of the Declaration of Helsinki.

### Imaging

Imaging occurred only after complete surgical removal of the initially implanted DBS System. Anatomical and diffusion tensor imaging was performed on a clinical 3-Tesla MRI system (Siemens Magnetom Trio Tim System 3 T, Erlangen, Germany). Anatomical sequences: 12-channel head coil, 3D MPRAGE (Magnetisation Prepared Rapid Gradient Echo): TR 390 ms, TE 2.15 ms, TI 800 ms, Flip angle 15°, voxel size 1.0 × 1.0 × 1.0 mm^3^, acquisition time 3:15 min. 3D T2 SPACE-sequence: TR 2500 ms, TE 231 ms, echo train length 141, flip-angle variable, voxel size 1.0 × 1.0 × 1.0 mm^3^, acquisition time 6:42. Diffusion tensor imaging: Single shot 2D SE EPI, TR 10,000 ms, TE 94 ms, diffusion values b = 0 s/mm^2^, b = 1000 s/mm^2^, diffusions directions 61, slice count 69, voxel size 2.0 × 2.0 × 2.0 mm^3^, acquisition time 11:40. Deformation correction of the EPI sequence according to Zaitsev et al. 2004 [24]. Postoperative imaging was performed with helical computed tomography (hCT).

### Fiber tracking and surgical planning

Deterministic FT was performed on a Linux workstation using StealthViz DTI (Version2, Medtronic Navigation, Louisville, CO, USA). We have preciously described fiber tracking of the cerebello-thalamo-cortical network (DRT) and surrounding structures (CST, ml) [1, 3, 4, 7] for the purpose of DTI FT-assisted DBS surgery. We used the Framelink 5.0 software (Medtronic SNT, Louisville, USA) for trajectory planning. The anatomical data including the generated DTI data were uploaded to the planning system. It was made sure that the beneficial initial electrode position was part of the newly planned trajectory. The new target point was planned into the center of the DTI-derived DRT in the subthalamic region (STR) roughly at the position of the caudal zona incerta (cZI) [16]. Chosen trajectories excluded vessels, sulci, and ventricular structures.

#### Surgery

**Fig. 1:**
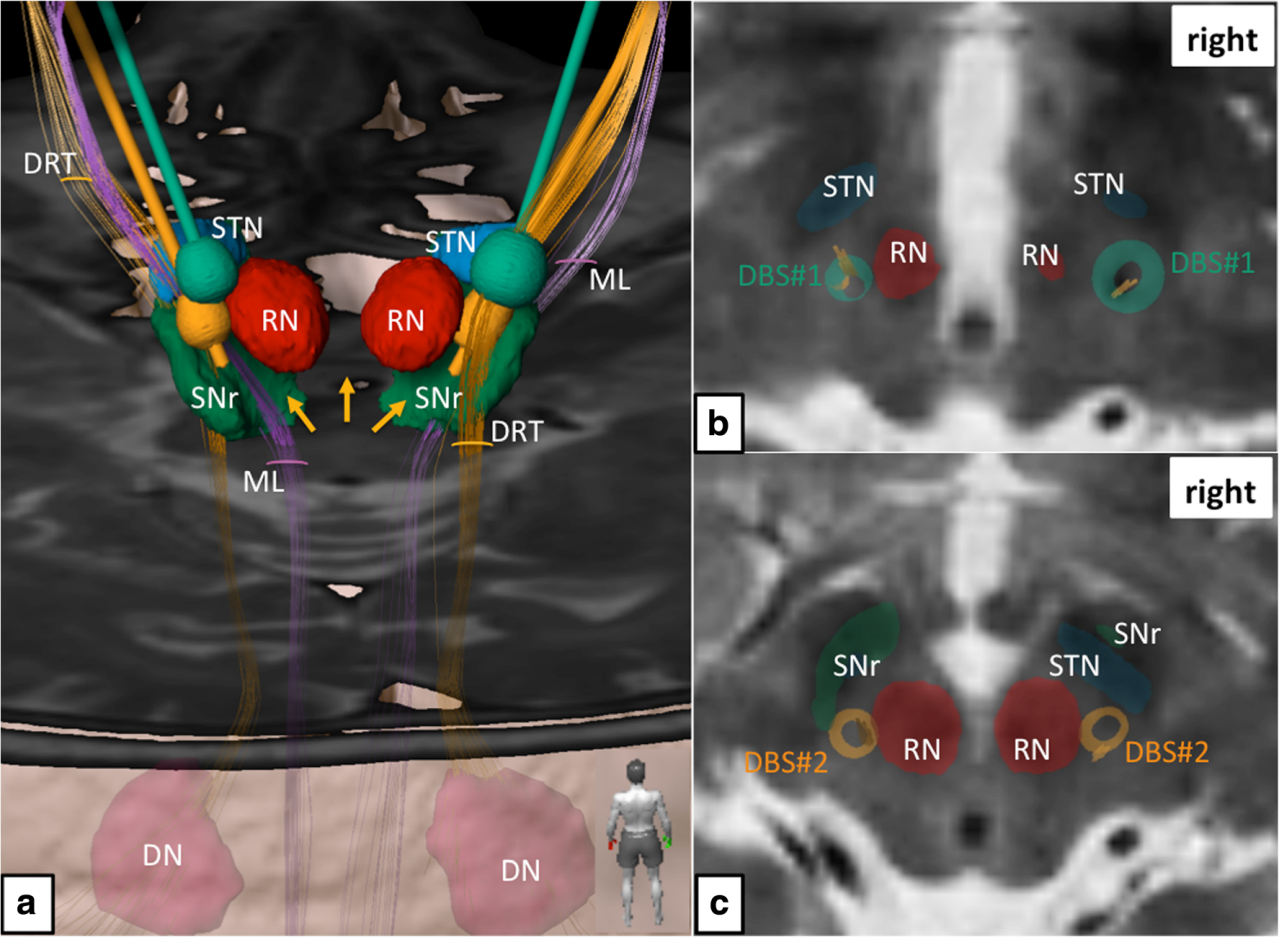
Simulation of primary DBS surgery (targeting the Vim nucleus; green electrodes and electric fields) and revision surgery (targeting the subthalamic DRT; yellow electrodes and electric fields). **a** Three-dimensional rendition as viewed from posterior. Electric fields in both situations show clear and bilateral involvements of the DRT. Left revision surgery shows proximity to the medial lemniscus (ML). *Yellow arrows* indicate missing depiction of the DRT’s crossing, which is a downside of the deterministic tracking algorithm. **b** Thalamic level (roughly at level of MCP) shows coverage of DRT fibers (*yellow*) by the electric fields (*green*) of initial DBS surgery. **c** Subthalamically the electric fields after revision surgery (*yellow*) are smaller but still cover the entire DRT. Legend: *DN* = dentate nucleus, *DRT* = dentate-rubro-thalamic tract, *ML* = medial lemniscus, *RN* = red nucleus, *SNr* = substantia nigra, *STN* = subthalamic nucleus

The initially implanted Medtronic DBS system was completely removed in order to allow for 3-Tesla MRI. Four weeks after this surgical removal, DBS revision surgery was performed. In order to allow for stimulation in the thalamic and subthalamic region, we elected for an octopolar linear lead (15-mm active tip, eight contacts, Boston Scientific, MA, USA). We planned an approach that would allow stimulating the initially stimulated region by placing contacts at the MCP level in the same position as during DBS#1 but extending to the subthalamic region (cf. Table 1). The trajectory thus includes the rather classical Vim-region but extends into the caudal zona incerta (cZi) inferiorly (cf. Fig. 1c) by this penetrating the DRT on its way between cZI and Vim on its full length. We have previously described our targeting approach in more detail [3].

**Table 1:**
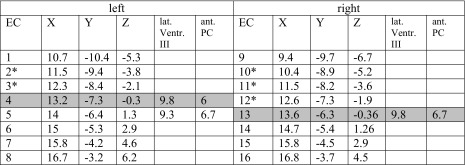
Mid-commissural point (MCP) coordinates of DBS electrode contacts (EC), after revision surgery (DBS#2)

Legend: grey shading = approximate level of MCP, these coordinates represent also position of active contacts during DBS#1; *negative Z* = below MCP; * = activated (cathodal) contacts; *Ventr. III* = 3rd ventricle; *EC* = electrode contact; *ant.* = anterior; *lat.* = lateral; *PC* = posterior commissure; note: 1 and 9 are the most inferior, 8 and 16 are the most superficial contacts

Stereotactic placement of the DBS leads was performed with a Leksell G-Frame (Elekta, Sweden) with the patient under local anesthesia. Intraoperative testing of the DRT target region was performed using a Cosman lesion generator/stimulator (Cosman, USA) and a 2-mm exposed rod electrode that was introduced over a micro drive (FHC, Bowdoin, USA). The electrode was advanced from 6 mm above the target region (2 mm above MCP) in 2-mm steps into the target region. Only extremity tremor could be tested on the OR table. Test stimulation looked for an acceptable threshold of effects and side effects and symptom control. After bilateral electrode placement, the Vercise PC rechargeable generator (Boston Scientific, CA, USA) was surgically placed under the clavicle under general anesthesia on the same day.

#### Programming after revision surgery

After settling of the initial microlesioning effect stimulation was initiated starting with a monopolar review. Stimulation focused in the contacts in the subthalamic region (cf. Table 1). Long-term stimulation is: channel 1, left, case +, 2-(80%), 3-(20%), 3 mA, 60 µs, 174Hz; channel 2, right, case +, 10- (40%), 11-(40%), 12-(20%), 3.5 mA, 60us, 174 Hz. Initially, the patient was stimulated with a somewhat different and less complex setting until the microlesioning effect vanished (channel 1, left, case+, 2-, 3 mA, 60 µs, 174 Hz; channel 2, right, case+, 11- (47%), 12- (5%), 3 mA, 60 µs, 174 Hz). Over time, the contacts changed and the pulse width was reduced to 30 µs in order to reduce side effects and increase therapeutic width [19]. The settings have now been stable for over 6 months.

#### Postoperative imaging workup

DBS leads, stimulation fields, and fibers were reconstructed using the Brainlab Elements software for cranial surgery planning (Brainlab AG, Feldkirchen, Germany) and Boston Scientific GUIDE XT software (Boston Scientific Corp., Boston, MA, USA).

**Fig. 2:**
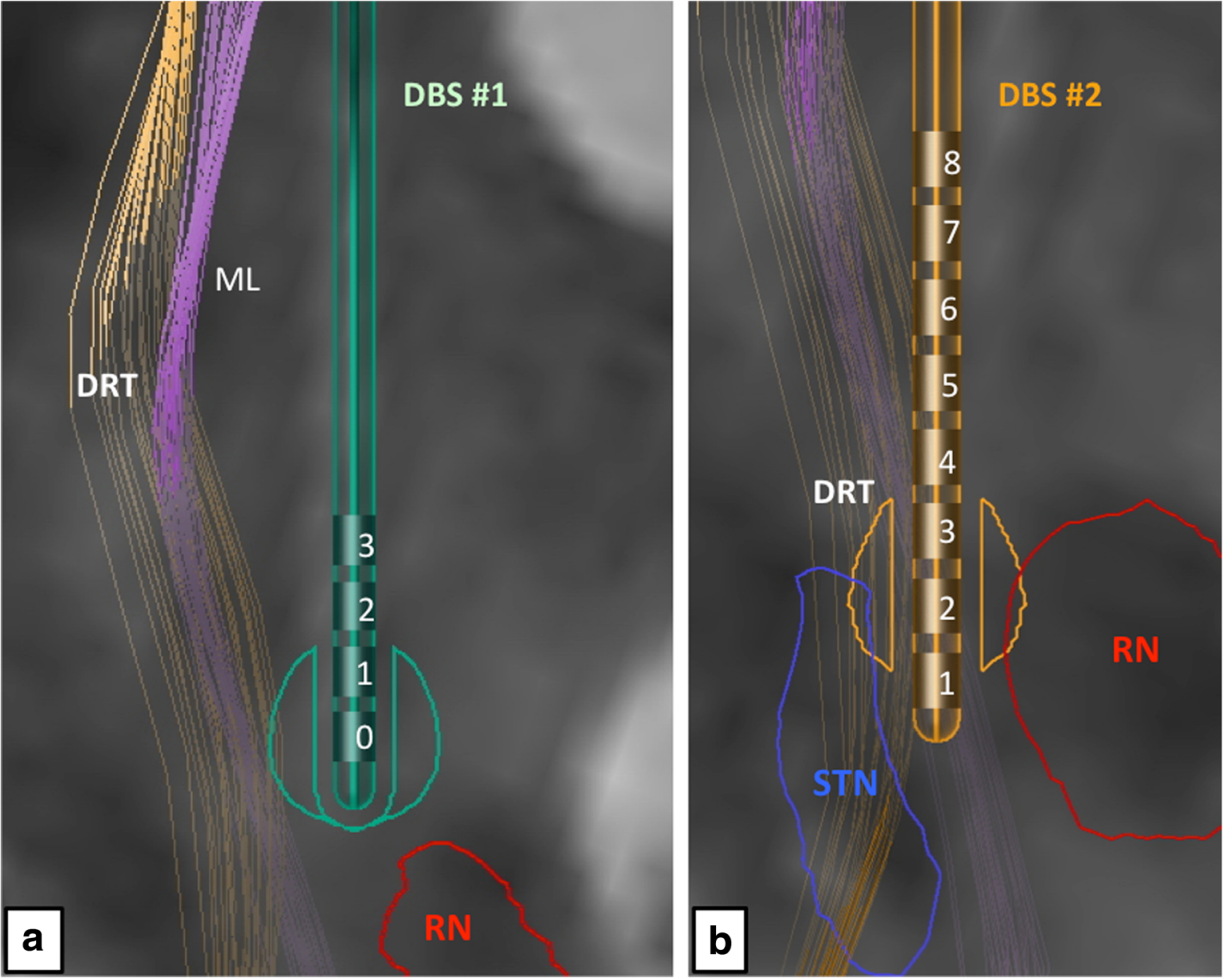
Simulation of (*left*) electrode positions in their functional environment. **a** Initial DBS surgery and slightly eccentric position of the electric field, superficial to the red nucleus (RN) level. **b** Revision surgery. The tip of the electrode is intercalated between posterior STN and RN (for legend, cf. Fig. 1)

The trajectories of the implanted electrodes have been reconstructed with an automatic detection algorithm based on the respective post-OP CT. Both postoperative CTs (after DBS#1 and DBS#2, respectively) have been fused with the highest-resolution pre-operative MR imaging in order to display all four trajectories co-localized in the tensor space used for fiber tractography (tensors reconstructed using B-vector realignment, Rician de-noising and Eddy CurrentCorrection). The leads were modeled in 3D, based on the characteristic profile of the Boston Scientific DB-2201 lead and the Medtronic 3389 lead, respectively (cf. Figs. 1 and 2).

For each lead, the volume of tissue activated induced by the stimulation fields was simulated based on the applied stimulation parameter settings. In DBS#1, we used a simplified model of simulation to create a sphere-shaped EF [14]. In DBS#2, simulation was performed using the GUIDE XT software.

For DBS#1 (Medtronic 3389 lead), the following stimulation settings were used for EF simulation: left side (quadripolar, EC0-3, EC0 most distal), EC0 2.4 V(−), pulse width 150 μs; right side (quadripolar, EC8-11, EC8 most distal), EC8 3.5 V(−), pulse width 60 μs; both with a rate of 210 Hz.

For DBS#2 (Boston DB-2201 lead), the following stimulation settings were used for EF simulation: left side (octopolar, effective contacts (EC) 1–8, EC1 most distal), EC2 2.6 mA(−) and EC3 0.6 mA(−); right side (octopolar, EC9-16, EC9 most distal), EC10 1.4 mA(−), EC11 1.4 mA(−), EC12 0.8 mA(−); both with a frequency of 174 Hz and a pulse width of 30 μs.

The clinical DTI datasets serving as a basis for the fiber reconstruction were corrected for distortion effects by means of patch-wise affine registration and interpolating multiple registration estimates in order to calculate a deformation field for the entire DTI volume. Deterministic fiber tracking (fractional anisotropy = 0.20, minimum fiber length = 20 mm, maximal curvature = 90°) was used to reconstruct the fibers that are enclosed by the respective stimulation field. The dentato-rubro-thalamic tract (DRT) was reconstructed independent of respective lead position or stimulation settings and is displayed for comparison (Fig. 1). DRT reconstruction was based on a tractographic reconstruction of fibers ascending the fourth ventricle wall dorsolaterally (superior cerebellar peduncle), crossing the midbrain area and passing the vicinity of the RN and the Vim, ending in the ipsilateral motor cortex (prcg). It is of note that typical for the deterministic algorithm used here, the crossing of the fibers below the RN could not be visualized (cf. Fig. 1, yellow arrows).

**Fig. 3:**
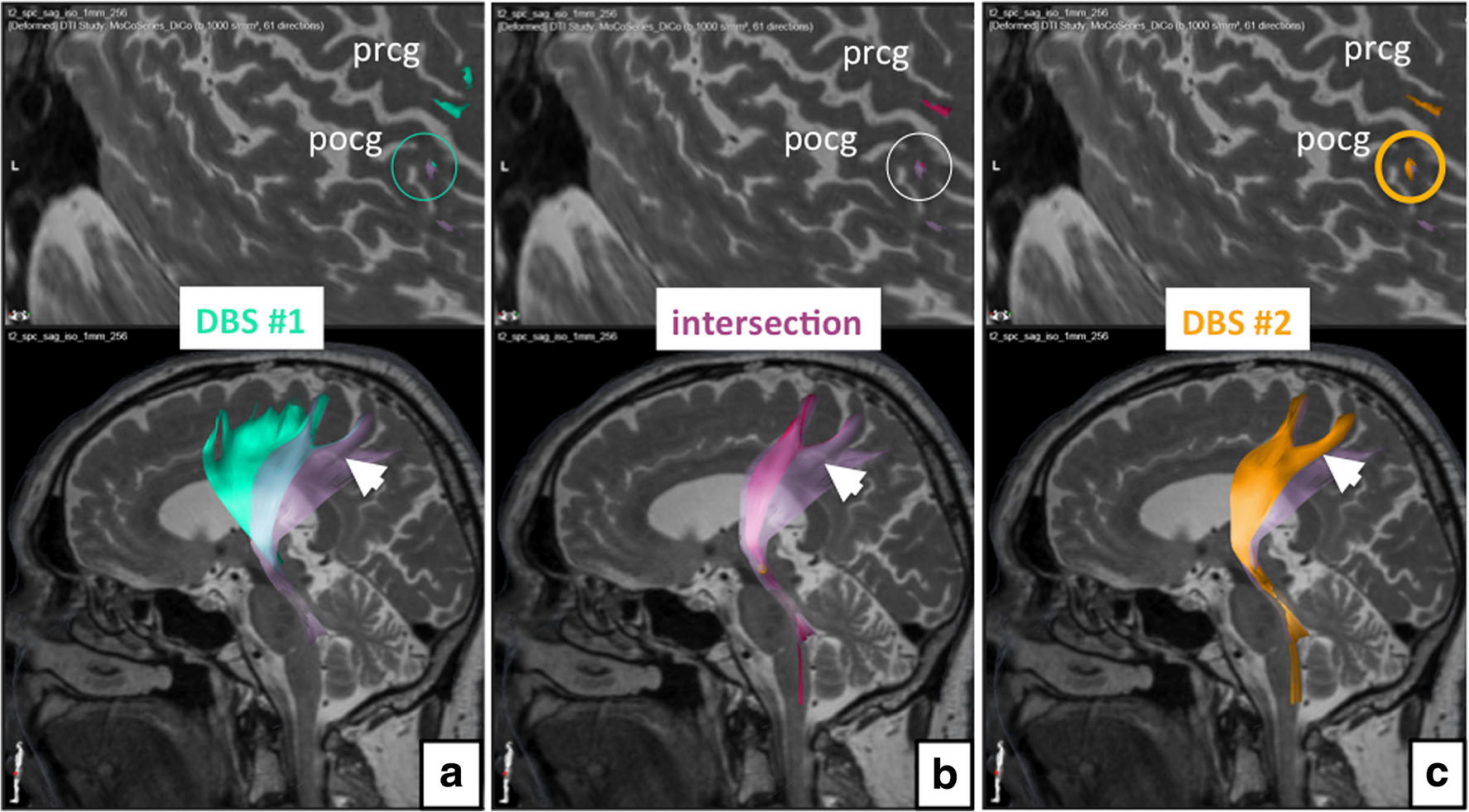
Analysis of the distant cortical connections in initial DBS (**a**) and under conditions of revised DBS (**c**). The *upper row* shows the individual curved surface or Mercator projections for the cortical region (only left side shown). **b** The fiber tracts involved in both stimulation situations. Initial DBS obviously shows a less selective activation of fibers projecting to dorsal prefrontal rand supplementary motor region and a lesser focus on typical DRT fibers. The revision DBS for this left side obviously shows a stronger involvement of the postcentral gyrus (pocg)

**Fig. 4:**
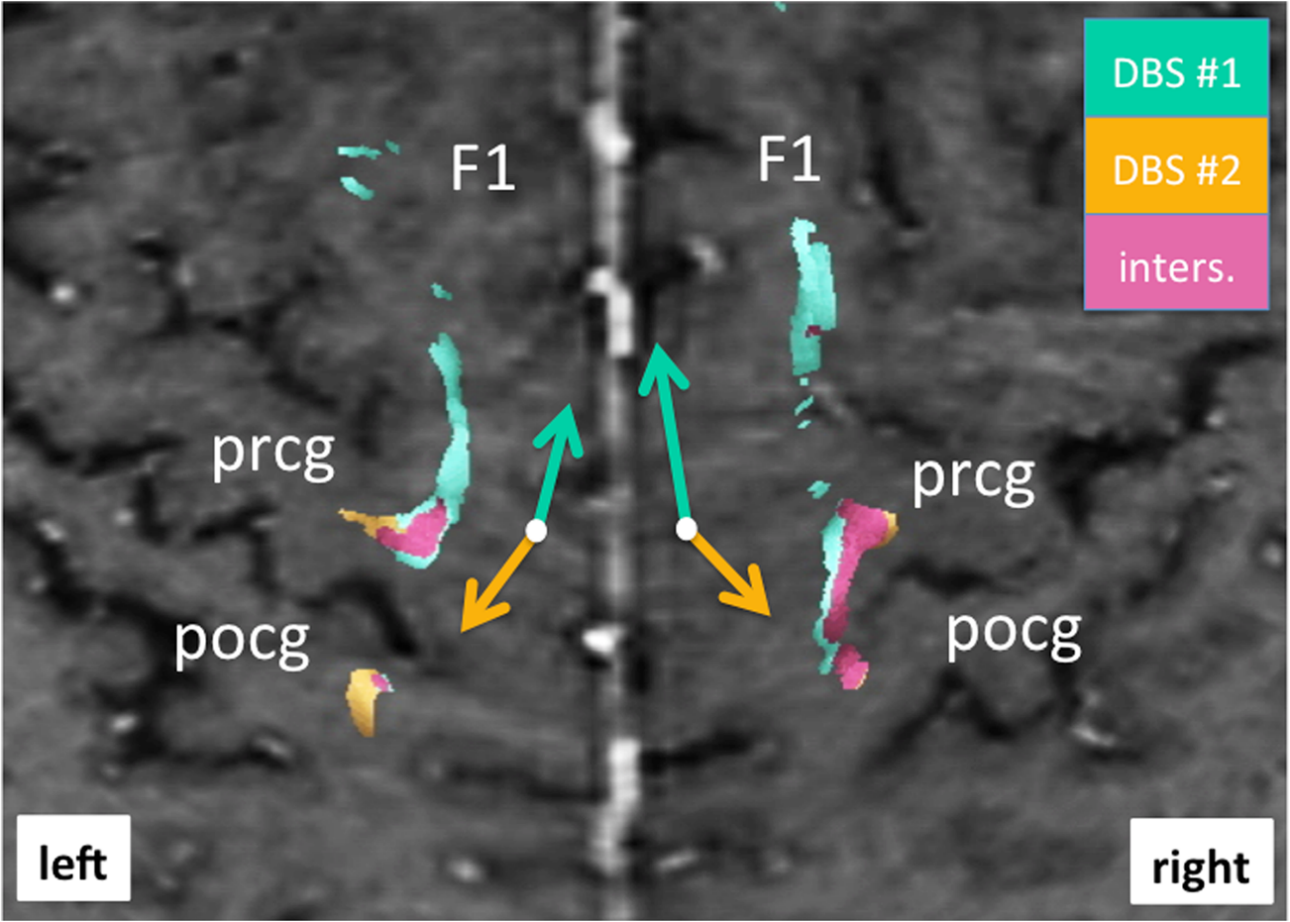
Bilateral curved surface or Mercator projection showing the cortical connections of fiber tracts involved with DBS. Note how revision surgery (DBS#2, *yellow*) shows a more posterior and lateral cortical connection (*yellow arrow*) focusing more on the precentral gyrus (prcg) and (to a lesser extent) postcentral gyrus (pcog). Projections form the initial DBS surgery (DBS#1, *green*) project more anteriorly and medially (as indicated by *green arrows*) (for legend cf. Fig. 3)

In order to better understand the differentially involved fiber pathway projections in DBS#1 and DBS#2 we used the electric field simulation models as seed volumes (VOI, inclusive) for another round of deterministic tracking. We also defined the fiber bundles that were effectively involved in both situations (cf. Figs. 3 and 4). In order to better appreciate the projection pattern of the different fiber pathways activated, we use a Mercator projection of the cortical surface [22] (cf. Figs. 3 and 4).

## Results

Clinically, trunk and extremity tremor markedly improved immediately after stimulation initiation (settings, see above). The preoperative ETRS 51 (before DBS#2) improved to a postoperative ETRS score of 20 (61% improvement, for sub-scores cf. Table 2). The patient developed moderate dysarthria and gait stability problems, which were treated with physiotherapy and speech therapy. An adjustment of the pulse width to 30 µs ameliorated these problems. The improvement is sustained after 1 year of stimulation.

**Table 2:**
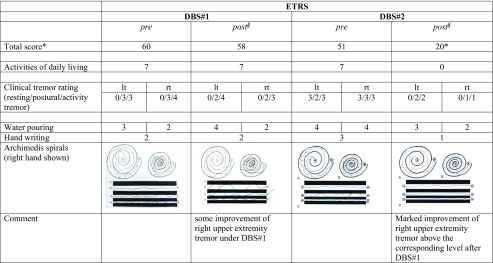
Tremor ratings

*Total score does NOT represent the sum of the here only selectively presented sub-items, *lt* = left, *rt* = right

§ under stimulation, respectively; some degree of worsening between DBS#1 postop and DBS#2 preop is likely due to termination of stimulation upon removal oft he DBS system. *marked improvement of total ETRS

The detailed neuroimaging evaluation revealed the involvement of the DRT in both implantation instances (cf. Fig. 1). Using the individually simulated elective fields (EFs) as seed volumes helped to better understand the difference between the implantation with respect to cortical fiber projections. In the first implantation (DBS#1), more anteriorly located projections were included indicating more diffuse activation of fiber tracts traversing the VOp (ventralis oralis posterior nucleus) and possibly VOa (ventralis oralis anterior nucleus) and ascending to the supplementary motor region (cf. Fig. 3). Both nuclei are not visible on MRI sequences. In the second implantation (DBS#2), more posteriorly projecting fibers are included. These fibers also focused more densely on the DRT projections including more laterally projecting fiber bundles. As a summary: As opposed to DBS#1, the pattern of modulated fiber pathways shifted more posterior-laterally in the cortical projection (cf. Fig. 4).

## Discussion

The clinical case presented here benefited from a revision surgery that was performed with DTI FT assistance. It is known that the subthalamic region might be more beneficial for the treatment of more complex forms of tremor [11, 16, 17]. Probably the choice of a thalamic target (Vim) in the light of extensive and debilitating trunk tremor was suboptimal in the first place. According to our understanding, the cZI and the STR represent parts of the DRT network [3, 4] that can be accessed at any given point, even post-synaptically above the MCP level [4, 10]. We used the DTI-FT-assisted approach to be able to promise a more successful revision surgery. According to the idea that was operationalized by Plaha [17], we planned to stimulate fibers right below the entrance of the cerebello-thalamic projections into the Vim nucleus. This position should in principle allow stimulating the DRT more completely (including also the fibers related to trunk tremor control) with lesser current allowing for a better tremor control with lesser side effects. In this respect, the complete DRT has to be covered by an EF.

The neuroimaging work-up that we were able to perform allowed verifying most of these assumptions. We were able to show that the DRT was part of both stimulations but when looking at the fiber projections in comparison of DBS#1 and DBS#2 (based on the EF simulation) we found differential patterns of fiber projection based on the individually simulated EFs. Stimulation at the thalamic level during DBS#1 involved the DRT but was not able to cover the region densely enough and induced a modulation of more anteriorly and supplementary motor projection pathways. After revision surgery (DBS#2), we were able to stimulate a smaller EF, which was much more confined but also showed some side effects (dysarthria and gait disturbances) owing to the activation of projections to the pocg. We were able to circumvent these side effects by reducing the pulse width of the simulation [19] and by steering the current along the DRT (cf. Table 1).

### Limitations

The DTI FT technology, especially when using a deterministic algorithm, cannot always present resulting fiber bundles as true anatomical structures. This is especially true for the DRT as a double curved structure, which is inherently difficult to track for any algorithm. As a tribute to the limitations of the deterministic approach, we here accept the fact that the crossing below the RN level cannot be shown. We are, however, confident, that we target a structure that is accurately depicted in the part that we aim for. This assumption is based on our experience with DTI FT in numerous patients over more than 6 years. Thus, except the crossing, which is missing, the targeted structure is accurately and anatomically sound depicted [2–4, 7].

The system that was used to evaluate the fiber tract projection (Elements, Brainlab, Feldkirchen, Germany) is different from the one that was used for the initial DTI-FT-assisted surgery (StealthViz DTI, Medtronic, USA). This should, within certain limitations introduced in the depiction of fiber pathways [1], make our approach more valid, although we do not use only deterministic tracking approaches in both systems. Certainly, we cannot acquire new DTI imaging after implantation of a DBS system and rely completely on preoperative imaging and the depiction of an electrode’s active contact by hCT.

We have to address that the simulation models for the EFs are different between the electrodes (DBS#1 vs. DBS#2) and that this could introduce some error. However, both models have been evaluated individually. They are necessary because of the individual electrode features.

Of another concern is the accuracy of the method that has to be addressed. We were recently able to investigate the accuracy of the DTI-FT approach for DBS and despite the results of other groups [20], concluded that the accuracy of the approach allows for direct targeting in DBS surgery [5, 6]. Other groups are now starting to use probabilistic tracking algorithms to resolve problems of inferior resolution, kissing, branching, and crossing fibers and the problem of deformed fibers [15]. At this moment, however, there is no clear indication that on the single case basis this is a valid approach next to the fact that commercially available and certified planning systems for surgical interventions are all based on deterministic algorithms.

## Conclusions

The DRT is an important tremor-reducing structure and can be directly targeted with DBS. In the presented case of initially failed thalamic DBS surgery for essential tremor, a clear involvement of the DRT but also more anteriorly projecting pathways could be shown. This explains the inferior efficacy of the primary implantation, retrospectively. Revision surgery was performed with DTI FT assistance and directly targeted for the DRT subthalamically. The rational was a possibly more focused modulation of the DRT, especially with respect to trunk and proximal extremity tremor control, which was actually achieved. Further analysis and comparison to initial surgery revealed a lateral and posterior shift of the modulation in the cortical fiber pathway projection pattern after revision surgery as compared to initial implantation. This pattern allows explaining a more efficacious stimulation after electrode replacement. Moreover, a different side-effects spectrum with more involvement of a postcentral projection (cf. Figs. 3 and 4), especially on the left side, after revision can readily be explained.

There are clear limitations especially of the deterministic DTI FT, anatomical ground truth and accuracy, which we have discussed. Nevertheless, our work underpins the importance of direct targeting using advanced neuroimaging technologies to perform personalized DBS surgery. The evaluation of DBS electrode positions with the herein-described technologies will in turn improve targeting and revision strategies. In the future, DBS surgery will likely be based on more solid neuroimaging information on a regular basis as this will become safer and will likely allow performing DBS under general anesthesia. Direct targeting with DTI FT-assisted approaches is the focus of our ongoing research in a variety of indications.

Abbreviations: cZI, Caudal zona incerta; DBS, Deep brain stimulation; DRT, Dentato-rubro-thalamic tract; DTI, Diffusion tensor magnetic resonance imaging; EC, Effective contact; ET, Essential tremor; F1, Superior frontal gyrus; FT, Fiber tractography; hCT, Helical computed tomography; MCP, Mid-commissural point; ML, Medial lemniscus; MRI, Magnetic resonance imaging; Pocg, Postcentral gyrus; Prcg, Precentral gyrus; pSTR, Posterior subthalamic region; RN, Red nucleus; STN, Subthalamic nucleus; SNr, Substantia nigra pars resticulata; Vim, Ventral intermediate nucleus of thalamus; Voa, Ventralis oralis anterior nucleus of thalamus; Vop, Ventralis orialis posterior nucleus of thalamus.

## References

[1] Burgel U, Madler B, Honey CR, Thron A, Gilsbach J, Coenen VA (2009). Fiber tracking with distinct software tools results in a clear diversity in anatomical fiber tract portrayal. Cen Eur Neurosurg.

[2] Calabrese E (2016). Diffusion tractography in deep brain stimulation surgery: a review. Front Neuroanat.

[3] Coenen VA, Allert N, Madler B (2011). A role of diffusion tensor imaging fiber tracking in deep brain stimulation surgery: DBS of the dentato-rubro-thalamic tract (drt) for the treatment of therapy-refractory tremor. Acta Neurochir (Wien).

[4] Coenen VA, Allert N, Paus S, Kronenburger M, Urbach H, Madler B (2014). Modulation of the cerebello-thalamo-cortical network in thalamic deep brain stimulation for tremor: a diffusion tensor imaging study. Neurosurgery.

[5] Coenen VA, Fromm C, Kronenburger M, Rohde I, Reinacher PC, Becker R, Marks B, Gilsbach JM, Rohde V (2006). Electrophysiological proof of diffusion-weighted imaging-derived depiction of the deep-seated pyramidal tract in human. Zentralbl Neurochir.

[6] Coenen VA, Jenkner C, Honey CR, Madler B (2016). Electrophysiologic validation of diffusion tensor imaging tractography during deep brain stimulation surgery. AJNR Am J Neuroradiol.

[7] Coenen VA, Madler B, Schiffbauer H, Urbach H, Allert N (2011). Individual fiber anatomy of the subthalamic region revealed with diffusion tensor imaging: a concept to identify the deep brain stimulation target for tremor suppression. Neurosurgery.

[8] Deuschl G, Bain P, Brin M (1998). Consensus statement of the Movement Disorder Society on Tremor. Ad Hoc Scientific Committee. Mov Disord.

[9] Elias WJ, Lipsman N, Ondo WG, Ghanouni P, Kim YG, Lee W, Schwartz M, Hynynen K, Lozano AM, Shah BB, Huss D, Dallapiazza RF, Gwinn R, Witt J, Ro S, Eisenberg HM, Fishman PS, Gandhi D, Halpern CH, Chuang R, Butts Pauly K, Tierney TS, Hayes MT, Cosgrove GR, Yamaguchi T, Abe K, Taira T, Chang JW (2016). A randomized trial of focused ultrasound thalamotomy for essential tremor. N Engl J Med.

[10] Fasano A, Herzog J, Raethjen J, Rose FE, Muthuraman M, Volkmann J, Falk D, Elble R, Deuschl G (2010). Gait ataxia in essential tremor is differentially modulated by thalamic stimulation. Brain J Neurol.

[11] Hamel W, Herzog J, Kopper F, Pinsker M, Weinert D, Muller D, Krack P, Deuschl G, Mehdorn HM (2007). Deep brain stimulation in the subthalamic area is more effective than nucleus ventralis intermedius stimulation for bilateral intention tremor. Acta Neurochir (Wien).

[12] Henderson JM (2012). “Connectomic surgery”: diffusion tensor imaging (DTI) tractography as a targeting modality for surgical modulation of neural networks. Front Integr Neurosci.

[13] Lozano AM (2000). Vim thalamic stimulation for tremor. Arch Med Res.

[14] Madler B, Coenen VA (2012). Explaining clinical effects of deep brain stimulation through simplified target-specific modeling of the volume of activated tissue. AJNR Am J Neuroradiol.

[15] Petersen MV, Lund TE, Sunde N, Frandsen J, Rosendal F, Juul N, Ostergaard K (2016) Probabilistic versus deterministic tractography for delineation of the cortico-subthalamic hyperdirect pathway in patients with Parkinson disease selected for deep brain stimulation. J Neurosurg: 1–12 10.3171/2016.4.JNS1624 27392264

[16] Plaha P, Khan S, Gill SS (2008). Bilateral stimulation of the caudal zona incerta nucleus for tremor control. J Neurol Neurosurg Psychiatry.

[17] Plaha P, Patel NK, Gill SS (2004). Stimulation of the subthalamic region for essential tremor. J Neurosurg.

[18] Rehncrona S, Johnels B, Widner H, Tornqvist AL, Hariz M, Sydow O (2003). Long-term efficacy of thalamic deep brain stimulation for tremor: double-blind assessments. Mov Disord.

[19] Reich MM, Steigerwald F, Sawalhe AD, Reese R, Gunalan K, Johannes S, Nickl R, Matthies C, McIntyre CC, Volkmann J (2015). Short pulse width widens the therapeutic window of subthalamic neurostimulation. Ann Clin Transl Neurol.

[20] Said N, Elias WJ, Raghavan P, Cupino A, Tustison N, Frysinger R, Patrie J, Xin W, Wintermark M (2014). Correlation of diffusion tensor tractography and intraoperative macrostimulation during deep brain stimulation for Parkinson disease. J Neurosurg.

[21] Sammartino F, Krishna V, King NK, Lozano AM, Schwartz ML, Huang Y, Hodaie M (2016). Tractography-based ventral intermediate nucleus targeting: novel methodology and intraoperative validation. Mov Disord.

[22] Scheef L, Hoenig K, Urbach H, Kuhl C, Schild H, Koenig R (2003). Curved-surface projection: an alternative method for visualizing functional MR imaging results. AJNR Am J Neuroradiol.

[23] Schlaier J, Anthofer J, Steib K, Fellner C, Rothenfusser E, Brawanski A, Lange M (2015). Deep brain stimulation for essential tremor: targeting the dentato-rubro-thalamic tract?. Neuromodulation.

[24] Zaitsev M, Hennig J, Speck O (2004). Point spread function mapping with parallel imaging techniques and high acceleration factors: fast, robust, and flexible method for echo-planar imaging distortion correction. Magn Reson Med.

